# The predictive values of monocyte–lymphocyte ratio in postoperative acute kidney injury and prognosis of patients with Stanford type A aortic dissection

**DOI:** 10.3389/fimmu.2023.1195421

**Published:** 2023-07-24

**Authors:** Yubin Chen, Kaiyi Dong, Cheng Fang, Hui Shi, Wenjie Luo, Can-e Tang, Fanyan Luo

**Affiliations:** ^1^ Department of Cardiac Surgery, Xiangya Hospital, Central South University, Changsha, Hunan, China; ^2^ Department of Endocrinology, Xiangya Hospital, Central South University, Changsha, Hunan, China; ^3^ The Institute of Medical Science Research, Xiangya Hospital, Central South University, Changsha, Hunan, China; ^4^ National Clinical Research Center for Geriatric Disorders, Xiangya Hospital, Central South University, Changsha, Hunan, China

**Keywords:** type A aortic dissection, monocyte-lymphocyte ratio, postoperative acute kidney injury, predictive model, risk factor

## Abstract

**Objectives:**

Postoperative acute kidney injury (pAKI) is a serious complication of Stanford type A aortic dissection (TAAD) surgery, which is significantly associated with the inflammatory response. This study aimed to explore the relationship between blood count-derived inflammatory markers (BCDIMs) and pAKI and to construct a predictive model for pAKI.

**Methods:**

Patients who underwent TAAD surgery were obtained from our center and the Medical Information Mart for Intensive Care (MIMIC)-IV database. The differences in preoperative BCDIMs and clinical outcomes of patients with and without pAKI were analyzed. Logistic regression was used to construct predictive models based on preoperative BCDIMs or white cell counts (WCCs). The performance of the BCDIMs and WCCs models was evaluated and compared using the receiver operating characteristic (ROC) curve, area under the ROC curve (AUC), Hosmer–Lemeshow test, calibration plot, net reclassification index (NRI), integrated discrimination improvement index (IDI), and decision curve analysis (DCA). The Kaplan–Meier curves were applied to compare the survival rate between different groups.

**Results:**

The overall incidence of pAKI in patients who underwent TAAD surgery from our center was 48.63% (124/255). The presence of pAKI was associated with longer ventilation time, higher incidence of cerebral complications and postoperative hepatic dysfunction, and higher in-hospital mortality. The results of the logistic regression indicated that the monocyte–lymphocyte ratio (MLR) was an independent risk factor for pAKI. The BCDIMs model had good discriminating ability, predictive ability, and clinical utility. In addition, the performance of the BCDIMs model was significantly better than that of the WCCs model. Analysis of data from the MIMIC-IV database validated that MLR was an independent risk factor for pAKI and had predictive value for pAKI. Finally, data from the MIMIC-IV database demonstrated that patients with a high MLR had a significantly poor 28-day survival rate when compared to patients with a low MLR.

**Conclusion:**

Our study suggested that the MLR is an independent risk factor for pAKI. A predictive model based on BCDIMs had good discriminating ability, predictive ability, and clinical utility. Moreover, the performance of the BCDIMs model was significantly better than that of the WCCs model. Finally, a high MLR was significantly associated with poor short-term survival of patients who underwent TAAD surgery.

## Introduction

Aortic dissection (AD) is one of the most life-threatening cardiovascular diseases; it is caused by a tear of the aortic intimal layer and the separation/dissection of the aortic wall (1). According to the Oxford Vascular Study, AD is most common in those aged 65–75 years; the incidence of AD in this population is approximately 35 cases per 100,000 people per year (2). Sino-RAD, the first Registry of Aortic Dissection in China, revealed an earlier onset age of AD in China of approximately 50 years (3). The incidence of AD in the Chinese population is approximately 2.8 cases per 100,000 people per year (4). The mortality is extremely high in patients with AD not receiving any treatment (5). Therefore, early diagnosis and swift treatment are important for the survival of patients with AD.

Stanford type A aortic dissection (TAAD) involves the ascending aorta, which could result in fatal events, such as aortic rupture, tamponade, malperfusion, and aortic valve insufficiency (1). Swift surgical repair and/or replacement of the aorta are needed to save patients with TAAD (6). Although significant improvements have been made in the surgery of TAAD, the in-hospital mortality rate remains high at 27% according to the International Registry of Acute Aortic Dissection (IRAD) study (6). Surgical complications, such as acute kidney injury (AKI), are closely related to the poor prognosis of patients who underwent TAAD surgery (7). Insufficient tissue perfusion, stress response, oxidative stress, and systematic inflammation caused by TAAD and surgery can contribute to the onset of postoperative AKI (pAKI) (8, 9). Zhang et al. (10) and Wang et al. (11) reported that pAKI was significantly associated with worse short-term and long-term mortality of patients who underwent TAAD surgery. Therefore, further exploration of risk factors for pAKI and construction of a predictive model for pAKI based on these risk factors are needed to predict pAKI at an early stage.

Nowadays, blood count-derived inflammatory markers (BCDIMs), including neutrophil–lymphocyte ratio (NLR), monocyte–lymphocyte ratio (MLR), platelet–lymphocyte ratio (PLR), systemic inflammation response index (SIRI), and systemic inflammation index (SII), were found to be significantly correlated with systematic inflammation (12). Studies have indicated that these inflammatory markers could be predictive factors of the prognosis of cardiovascular diseases including ischemic heart disease and TAAD (13–15). However, there are few studies focusing on the relationship between BCDIMs and pAKI. Considering that systematic inflammation is involved in the onset of pAKI (9, 16), we speculated that these inflammatory markers might be risk factors for pAKI and could predict pAKI onset.

In this study, the relationship between BCDIMs and pAKI was analyzed using data from our center and the Medical Information Mart for Intensive Care (MIMIC)-IV database. Then, two predictive models were constructed using BCDIMs and white cell counts (WCCs). Thereafter, the performance of the two predictive models was compared. Finally, the correlation between BCDIMs and the prognosis of patients who underwent TAAD surgery were analyzed.

## Materials and methods

### Study population

Consecutive patients with TAAD who underwent open-chest TAAD surgery at Xiangya Hospital, Central South University, from January 2016 to August 2022 were enrolled. The exclusion criteria were as follows: pregnancy, patients who died during surgery or within 24 h after surgery, patients with tumors, patients with chronic kidney diseases or chronic liver diseases, patients with previous cardiovascular surgery history, and patients with missing data. The study was conducted in accordance with the “Declaration of Helsinki”, and the Ethics Committee of Xiangya Hospital, Central South University approved this study. Written informed consent was waived because of the observational, retrospective nature of this study.

### Collection of data

Data of patients who underwent TAAD surgery were collected from the electronic medical record system of Xiangya Hospital, Central South University. Patients’ preoperative data included demographic variables, such as age, gender, weight, height, smoking history, drinking history, blood pressure at admission, and time from onset to surgery; underlying conditions, such as pregnancy, tumor, diabetes mellitus, hypertension, cardiac surgery history, chronic kidney diseases, chronic liver diseases, and Marfan syndrome; and laboratory tests, including white blood cell (WBC) count, neutrophil count, lymphocyte count, red blood cell (RBC) count, platelet count, hemoglobin, serum albumin, serum globulin, serum total bilirubin, serum direct bilirubin, serum alanine aminotransferase (ALT), serum aspartate aminotransferase (AST), serum urea, serum creatinine (SCr), serum uric acid, blood glucose, lactic acid, and international normalized ratio (INR). The operative data of patients included operation time, cardiopulmonary bypass time, aortic cross-clamp time, circulatory arrest time, surgical approach (total or semi-arch repair), cerebral protection strategy (antegrade or retrograde), circulatory arrest minimum temperature (anal temperature), and blood product usage intraoperation (including concentrated red blood cells, plasma, cryoprecipitate, and platelet). The postoperative data of patients included the arterial oxygen tension-inspired oxygen concentration (PaO_2_–FiO_2_) ratio, laboratory tests as mentioned above, delirium, spinal cord complications (including postoperative new-onset hemiplegia, paraplegia, and paraparesis), cerebral complications (including stroke and coma), and cardiac complications (including postoperative new-onset ventricular fibrillation, sudden cardiac arrest, and low cardiac output syndrome).

### Definition of BCDIMs, pAKI, hepatic dysfunction, and hypoxemia

For consistency, preoperative BCDIMs were calculated using the same measurement of the same blood sample. NLR, MLR, and PLR were defined as ratios of neutrophils, platelets, and monocytes to lymphocytes, respectively. SIRI was defined as neutrophils × monocytes/lymphocytes, and SII was defined as neutrophils × platelets/lymphocytes. Body mass index (BMI) was defined as weight/height^2^. The definition of pAKI was according to the criteria of the Kidney Disease: Improving Global Outcomes (KDIGO) (17). Briefly, pAKI was defined as a postoperative absolute increase in SCr level of ≥26.4 μmol/L within 48 h or ≥50% within 7 days of the initiation of renal replacement therapy after TAAD surgery. The baseline SCr level was defined as the first SCr test after admission. In this study, the urine output in the KDIGO criteria was not used because of the lack of urine output data from our center. Postoperative hepatic dysfunction was defined as the postoperative value of the Model of End-Stage Liver Disease (MELD) score higher than 12. The MELD score was calculated using the following standard formula: MELD score = 11.2 × ln (INR) + 3.78 × ln (serum total bilirubin [mg/dL]) + 9.57 × ln (SCr [mg/dL]) + 6.43 (18). Any variable with a value less than 1 was assigned a value of 1 to avoid a negative score, and values exceeding 4 for SCr were replaced by 4. The highest value of the MELD score within the first 24 h postoperatively was used to define postoperative hepatic dysfunction. Postoperative hypoxemia was defined as the value of the PaO_2_–FiO_2_ ratio lower than 300 mmHg (19). The highest value of the PaO_2_–FiO_2_ ratio within the first 24 h postoperatively was used to define postoperative hypoxemia.

### Construction of predictive models for pAKI

Univariate logistic regression analysis was used to screen variables for the predictive models. For the predictive model based on BCDIMs (BCDIMs model), age and preoperative (WCCs variables were not included) and operative variables with *p*-values of <0.10 were included. These variables were further screened by a variance inflation factor (VIF) and nomogram. Variables that were at risk of multicollinearity or contributed little to the model were excluded. After variable screening, the BCDIMs model was constructed by logistic regression *via* a stepwise enter method. A nomogram was then constructed based on the predictive model using the “rms” package in R software (version 4.2.3; R Foundation for Statistical Computing, Vienna, Austria). In addition, the predicted probability for pAKI of every patient was calculated according to the BCDIMs model for further analysis of the model performance.

For the predictive model based on WCCs (WCCs model), BCDIMs in the BCDIMs model were replaced with corresponding WCCs to construct the WCCs model. Other preoperative and operative variables were not changed. Then, the predicted probability for pAKI of every patient was calculated according to the WCCs model for further analysis of the model performance.

### Comparison of model performance

The performance of two predictive models was first evaluated by the discriminating ability using the receiver operating characteristic (ROC) curve and the area under the ROC curve (AUC). The ROC curves of two predictive models were generated, and the AUCs were then calculated using the “ROCR” package in R software (version 4.2.3). Thereafter, the difference between the AUC of the two predictive models was compared by the Delong method (20) using the “pROC” package in R software (version 4.2.3). Second, calibration was evaluated by the Hosmer–Lemeshow test and calibration plots, which could represent the relationship between observed probability and predicted probability derived from predictive models. The calibration plot was constructed using the “rms” package in R software (version 4.2.3), with the “boot” method using 1,000 replications. Third, the net reclassification index (NRI) and integrated discrimination improvement (IDI) index were further utilized to evaluate the additional predictive ability of the BCDIMs model compared to the WCCs model. The values of NRI and IDI were calculated, and their significance was analyzed using the “nricens” and “PredictABEL” packages in R software (version 4.2.3). Finally, decision curve analysis (DCA) was applied to compare the clinical utility of two predictive models using the “rmda” package in R software (version 4.2.3).

### Data extraction from the MIMIC-IV database

The MIMIC-IV database (version 2.0) is a large, longitudinal database that contains clinical data from the Beth Israel Deaconess Medical Center between 2008 and 2019. The first author of this study had completed the training and had credentialed access to the MIMIC-IV database. The data files of the MIMIC-IV database were downloaded from PhysioNet (https://physionet.org/content/mimiciv/2.0/). Then, the database for data extraction was constructed based on these files using PostgreSQL software (version 14, The PostgreSQL Global Development Group). Data of patients with TAAD and treated with open-chest surgery were extracted from the MIMIC-IV database. Duplicate patient data were excluded.

Patients who underwent TAAD surgery and had pAKI records in the MIMIC-IV database were extracted to analyze the relationship between pAKI and 1-year survival. After the screening, 147 patients underwent TAAD surgery and had pAKI records; the survival data of the 147 patients were obtained.

The preoperative variables of patients who underwent TAAD surgery and had pAKI records were further extracted to analyze the relationship between BCDIMs and pAKI. After the exclusion of patients with missing data, there were 72 patients extracted from the MIMIC-IV database for further analysis. In addition, the survival data of the 72 patients were obtained.

### Statistical analysis

Statistical analysis was performed using SPSS version 19 (IBM Corporation, Armonk, NY, USA) and R software (version 4.2.3). Continuous data were expressed as mean ± standard deviation (SD) or median, lower, and upper quartiles. Count data were expressed as frequency (percentage). Student’s *t*-test was used to compare the continuous data with normal distribution between different groups, and Mann–Whitney *U*-tests were applied to compare the continuous data with a non-normal distribution. For the count data, chi-square test was conducted to compare the difference in frequency between groups. Logistic regression was used to analyze the relationship between BCDIMs and pAKI and to construct the predictive model. The predictive value of the model was determined using ROC curves, AUC, NRI, and IDI. The Kaplan–Meier curve (log-rank test) was applied to compare the survival rate between different groups. A value of *p* < 0.05 was considered to be statistically significant.

## Results

### Characteristics of the study population and differences in BCDIMs between patients with and without pAKI

There were 457 patients with TAAD who underwent open-chest TAAD surgery in Xiangya Hospital, Central South University, from January 2016 to August 2022. According to the exclusion criteria, 202 patients were excluded: three patients with pregnancy, nine patients who died intraoperatively or within 24 h after surgery, 19 patients with chronic kidney or liver disease, 36 patients with previous cardiovascular surgery history, and 135 patients with missing data. Finally, 255 patients were included in this study.

**Figure 1:**
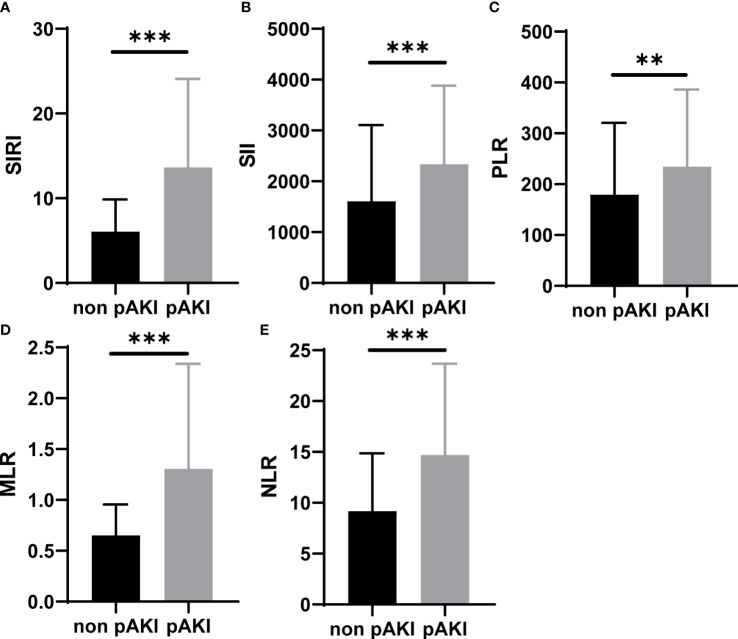
BCDIMs values in patients with or without pAKI from our center. **(A–E)** The values of SIRI, SII, PLR, MLR, and NLR in patients with or without pAKI. BCDIMs, blood count-derived inflammatory markers; pAKI, postoperative acute kidney injury; SIRI, systemic inflammation response index; SII, systemic inflammation index; PLR, platelet–lymphocyte ratio; MLR, monocyte–lymphocyte ratio; NLR, neutrophil–lymphocyte ratio. **p < 0.01, ***p < 0.001.

The preoperative and operative data of patients with TAAD are given in **Table 1**. The overall morbidity of pAKI in these patients was 48.63% (124/255). The mean age of patients without or with pAKI was 53.13 ± 12.56 and 50.96 ± 11.52 years, respectively. The proportion of men was significantly higher in the pAKI group (59.54% *vs.* 75.81%, *p* = 0.004). The mean BMI was significantly greater in the pAKI group (24.67 ± 3.80 *vs.* 26.04 ± 4.21 kg/m^2^, *p* = 0.001). The proportion of smokers in the pAKI group was significantly higher than in the non-pAKI group (48.09% *vs.* 60.48%, *p* = 0.031). In terms of laboratory variables, the neutrophil and monocyte counts were significantly increased in patients from the pAKI group (9.01 × 10^9^/L ± 3.55 *vs.* 10.40 ± 4.06 × 10^9^/L, *p* = 0.004; 0.71 ± 0.33 × 10^9^/L *vs.* 0.93 ± 0.47 × 10^9^/L, *p* < 0.001, respectively), while the lymphocyte count was significantly decreased in patients from the pAKI group (1.20 ± 0.56 × 10^9^/L *vs.* 0.92 ± 0.55 × 10^9^/L, *p* < 0.001). Moreover, the duration of the operation, cardiopulmonary bypass, and aortic cross-clamp in patients from the pAKI group was significantly greater than in patients from the non-pAKI group (407.63 ± 111.23 *vs.* 466.31 ± 115.42 min, *p* < 0.001; 196.27 ± 68.89 *vs.* 218.06 ± 66.65 min, *p* = 0.011; 106.18 ± 47.58 *vs.* 125.65 ± 82.45 min, *p* = 0.021, respectively). The proportion of patients who underwent total arch replacement was significantly higher in the pAKI group (80.15% *vs.* 90.32%, *p* = 0.034). The mean minimum temperature reached for circulatory arrest (CA) in patients from the pAKI group was significantly lower than that of patients from the non-pAKI group (26.12°C ± 2.56°C *vs.* 26.98°C ± 2.76°C, *p* = 0.010). Finally, BCDIMs were calculated and compared between different groups, and the results showed that the values of SIRI, SII, PLR, MLR, and NLR were significantly increased in patients from the pAKI group (**Figure 1**).

**Table 1:** Preoperative and operative variables of patients in different groups.

Variables	Non-pAKI group (n = 131)	pAKI group (n = 124)	*p*-Value
Age (years)	53.13 ± 12.56	50.96 ± 11.52	0.152
Gender (F/M)	53/78	30/94	**0.004**
BMI	24.67 ± 3.80	26.04 ± 4.21	**0.001**
Time from onset to surgery (h)	38 (24, 90)	40.5 (25.25, 71)	0.893
Acute AD (acute/subacute)	124/7	121/3	0.336
Hypertension (n (%))	88 (67.18)	94 (75.81)	0.083
Diabetes (n (%))	11 (8.40)	5 (4.03)	0.119
Marfan syndrome (n (%))	1 (0.86)	5 (4.03)	0.094
Smoking (n (%))	63 (48.09)	75 (60.48)	**0.031**
Drinking (n (%))	30 (22.90)	35 (28.23)	0.203
Systolic BP (mmHg)	135.63 ± 24.27	136.00 ± 27.81	0.909
Diastolic BP (mmHg)	65.36 ± 13.50	65.43 ± 16.06	0.971
White blood cells (×10^9^/L)	11.01 ± 3.79	12.36 ± 4.47	**0.009**
Red blood cells (×10^12^/L)	4.08 ± 0.56	4.13 ± 0.74	0.588
Hemoglobin (g/L)	122.86 ± 16.98	125.11 ± 22.82	0.371
Platelet (×10^9^/L)	179.90 ± 105.48	165.46 ± 57.38	0.179
MPV (fL)	9.81 ± 1.41	10.30 ± 8.03	0.487
Neutrophil (×10^9^/L)	9.01 ± 3.55	10.40 ± 4.06	**0.004**
Lymphocyte (×10^9^/L)	1.20 ± 0.56	0.92 ± 0.55	**<0.001**
Monocyte (×10^9^/L)	0.71 ± 0.33	0.93 ± 0.47	**<0.001**
Eosinophil (×10^9^/L)	0.05 ± 0.07	0.04 ± 0.07	0.269
RDW (%)	13.45 ± 1.19	13.56 ± 2.20	0.604
Albumin (g/L)	36.89 ± 4.78	37.53 ± 4.48	0.270
Globulin (g/L)	26.22 ± 3.85	25.67 ± 3.94	0.267
Total bilirubin (μmol/L)	19.36 ± 15.90	19.09 ± 11.00	0.874
Direct bilirubin (μmol/L)	7.63 ± 7.07	7.92 ± 5.01	0.708
ALT (U/L)	54.78 ± 113.99	68.55 ± 272.46	0.596
AST (U/L)	71.85 ± 153.91	80.97 ± 254.24	0.727
Urea (mmol/L)	10.13 ± 30.51	7.31 ± 4.10	0.307
Creatinine (μmol/L)	106.03 ± 70.61	134.38 ± 14.54	0.069
UA (μmol/L)	391.29 ± 138.61	374.15 ± 114.84	0.285
Blood glucose (mmol/L)	8.24 ± 3.31	7.91 ± 2.27	0.368
Lactic acid (mmol/L)	1.67 ± 1.40	1.53 ± 1.04	0.352
INR	1.14 ± 0.19	1.15 ± 0.17	0.746
Total arch replacement (n (%))	105 (80.15%)	112 (90.32%)	**0.034**
Operation time (min)	407.63 ± 111.23	466.31 ± 115.42	**<0.001**
Cerebral protection strategy (antegrade/retrograde)	113/18	114/10	0.165
Cardiopulmonary bypass time (min)	196.27 ± 68.89	218.06 ± 66.65	**0.011**
Aortic cross-clamp time (min)	106.18 ± 47.58	125.65 ± 82.45	**0.021**
Circulatory arrest time (min)	27.55 ± 21.64	26.66 ± 10.27	0.678
CA minimum temperature (°C)	26.98 ± 2.76	26.12 ± 2.56	**0.010**
CRBC usage intraoperation (U)	2.0 (0, 5.0)	3.5 (1.5, 5.375)	0.443
Plasma usage intraoperation (mL)	350 (0, 600)	400 (0, 600)	0.217
Cryoprecipitate usage intraoperation (U)	1.0 (0, 1.0)	1.0 (1.0, 1.0)	0.089
Platelet usage intraoperation (U)	1.0 (0, 1.0)	1.0 (0, 1.0)	0.092

p-Value in bold indicates that p-value is less than 0.05.

pAKI, postoperative acute kidney injury; BMI, body mass index; BP, blood pressure; MPV, mean platelet volume; RDW, red blood cell volume distribution width; ALT, alanine aminotransferase; AST, aspartate aminotransferase; UA, uric acid; INR, international normalized ratio; CA, circulatory arrest; CRBC, concentrated red blood cells.

### Clinical outcomes of patients with and without pAKI

**Figure 2:**
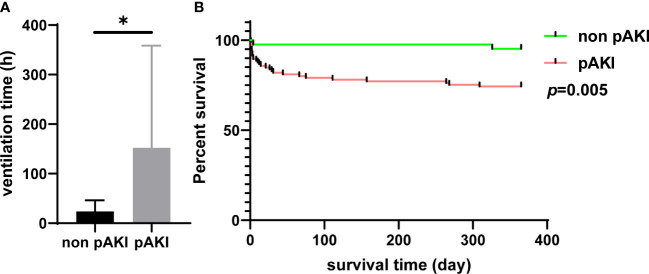
The relationship between pAKI and clinical outcomes of patients who underwent TAAD surgery from the MIMIC-IV database. **(A)** The mean postoperative ventilation time of patients with or without pAKI. **(B)** The 1-year Kaplan–Meier curve of patients with or without pAKI. pAKI, postoperative acute kidney injury; TAAD, type A aortic dissection; MIMIC-IV database, the Medical Information Mart for Intensive Care (MIMIC)-IV database. *p < 0.05.

According to data from our center, the proportions of patients with postoperative cerebral complications and hepatic dysfunction were significantly higher in the pAKI group (0.76% *vs.* 6.45%, *p* = 0.015; 55.73% *vs.* 92.74%, *p* < 0.001, respectively; **Table 2**). The in-hospital mortality rate was also significantly higher in the pAKI group compared to the non-pAKI group (0.76% *vs.* 6.45%, *p* = 0.015; **Table 2**). Data of patients from the MIMIC-IV database were further extracted to investigate the effects of pAKI on the clinical outcomes of patients. The results revealed that the ventilation time of patients from the pAKI group was significantly longer than that of patients from the non-pAKI group (**Figure 2A**). The Kaplan–Meier curve demonstrated that the 1-year survival rate of patients from the pAKI group was significantly lower than in patients from the non-pAKI group (**Figure 2B**). Overall, these results suggested that pAKI was closely associated with poor clinical outcomes in patients who underwent TAAD surgery.

**Table 2:** Clinical outcomes of patients in different groups.

Outcomes	Non-pAKI group (n = 131) (n (%))	pAKI group (n = 124) (n (%))	*p*-Value
Delirium	8 (6.11)	15 (12.10)	0.073
Cardiac complications	5 (3.82)	11(8.87)	0.079
Spinal cord complications	2 (1.53)	4 (3.23)	0.316
Cerebral complications	1 (0.76)	8 (6.45)	**0.015**
Hypoxemia	102 (77.86)	107 (86.29)	0.056
Hepatic dysfunction	73 (55.73)	115 (92.74)	**<0.001**
In-hospital mortality	1 (0.76)	8 (6.45)	**0.015**

p-Value in bold indicates that p-value is less than 0.05.

pAKI, postoperative acute kidney injury.

### Construction of a predictive model and nomogram for pAKI

To construct the predictive model, univariate logistic regression analysis was used to screen variables with *p*-values of <0.10 (**Supplementary Table 1**). First, age and preoperative and operative variables with *p*-values of <0.10 (except for blood count variables) were selected for the BCDIMs model. However, the results of the VIF indicated that the BCDIMs variables (including SIRI, SII, MLR, and PLR) and operative variables (including operation time and cardiopulmonary bypass time) might be at risk of multicollinearity (**Supplementary Table 2**). To avoid multicollinearity, we only kept the MLR and operation time in the predictive model, both of which had smaller VIF values. Then, according to the nomogram, we noticed that gender, age, and platelet usage intraoperation had little contribution to the model (**Supplementary Figure 1**). Therefore, gender, age, and operation time were excluded from the model.

**Figure 3:**
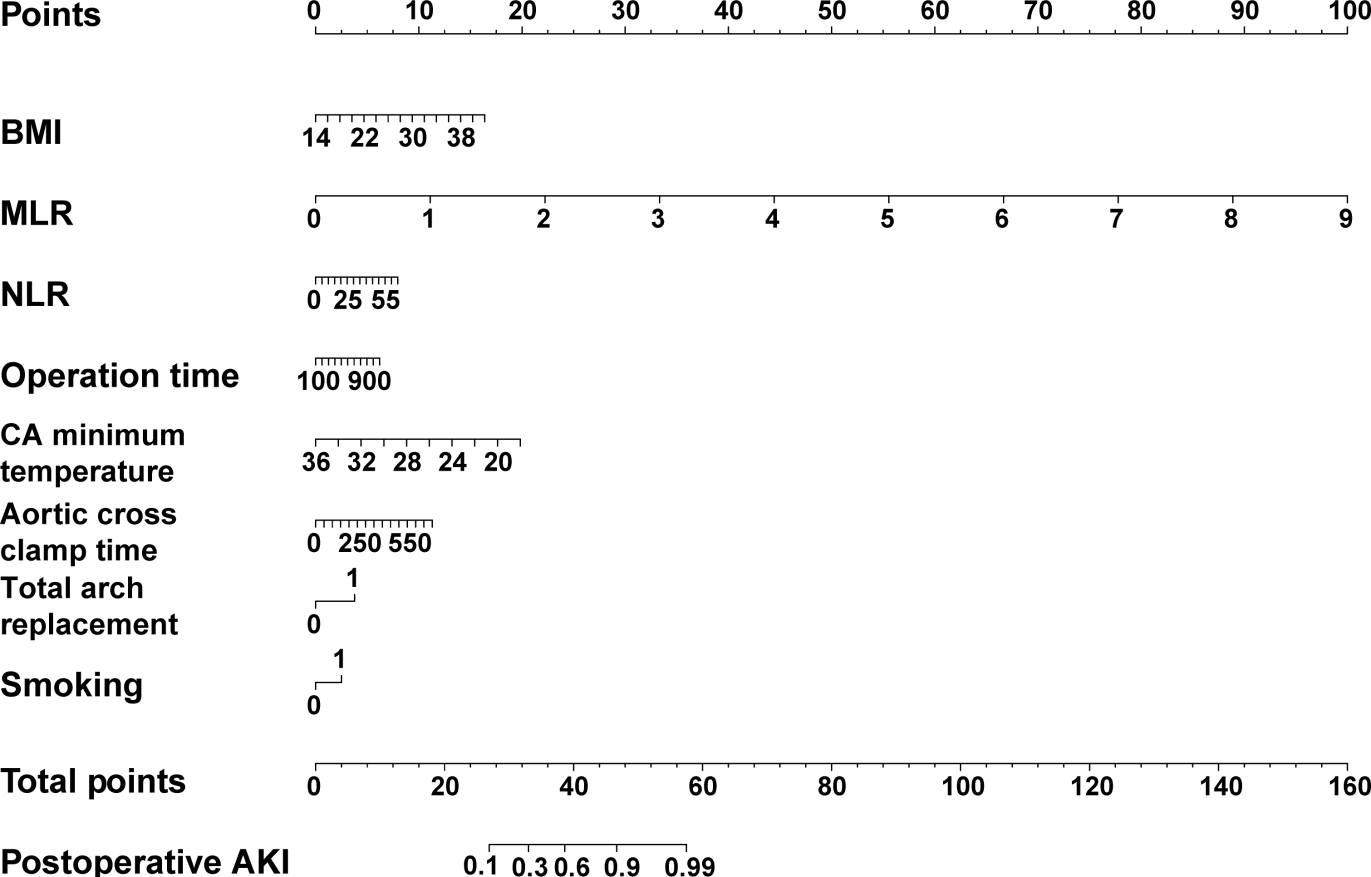
The nomogram of the predictive model based on BCDIMs. In the nomogram, gender 0 represents female, gender 1 represents male, smoking 0 represents non-smoker, and smoking 1 represents smoker. BCDIMs, blood count-derived inflammatory markers; BMI, body mass index; NLR, neutrophil–lymphocyte ratio; MLR, monocyte–lymphocyte ratio; AKI, acute kidney injury.

Finally, BMI, smoking status (smoker), MLR, NLR, total arch replacement, CA minimum temperature, operation time, and aortic cross-clamp time were selected to construct the BCDIMs model (**Table 3**). The results of the logistic regression analysis demonstrated that BMI, MLR, and CA minimum temperature were independent risk factors for pAKI (**Table 3**). A nomogram based on the predictive model was then constructed to visualize the prediction of pAKI (**Figure 3**).

**Table 3:** Logistic regression model for postoperative AKI (BCDIMs model).

Variables	β	OR	95% CI	*p*-Value
BMI	0.130	1.139	1.048–1.238	**0.002**
Smoking (smoker)	0.556	1.743	0.930–3.267	0.083
MLR	2.470	11.822	4.695–29.770	**<0.001**
NLR	0.027	1.028	0.971–1.087	0.346
Total arch replacement (yes)	0.838	2.312	0.809–6.605	0.118
CA minimum temperature	−0.245	0.783	0.680–0.902	**0.001**
Operation time	0.001	1.001	0.998–1.005	0.435
Aortic cross-clamp time	0.004	1.004	0.998–1.009	0.199

p-Value in bold indicates that p-value is less than 0.05.

AKI, acute kidney injury; BCDIMs, blood count-derived inflammatory markers; BMI, body mass index; MLR, monocyte–lymphocyte ratio; NLR, neutrophil–lymphocyte ratio; CA, circulatory arrest.

### Comparing the performance of the BCDIMs model and WCCs model

Next, we further evaluated the performance of the BCDIMs and compared it to the performance of the predictive model based on the WCCs variables. The BCDIMs variables in BCDIMs were replaced with corresponding WCCs variables. Briefly, the MLR and NLR were replaced with preoperative counts of monocytes, lymphocytes, and neutrophils. The WCCs model was then constructed, and the results suggested that BMI, lymphocytes, monocytes, and CA minimum temperature were independent risk factors for pAKI (**Supplementary Table 3**).

**Figure 4:**
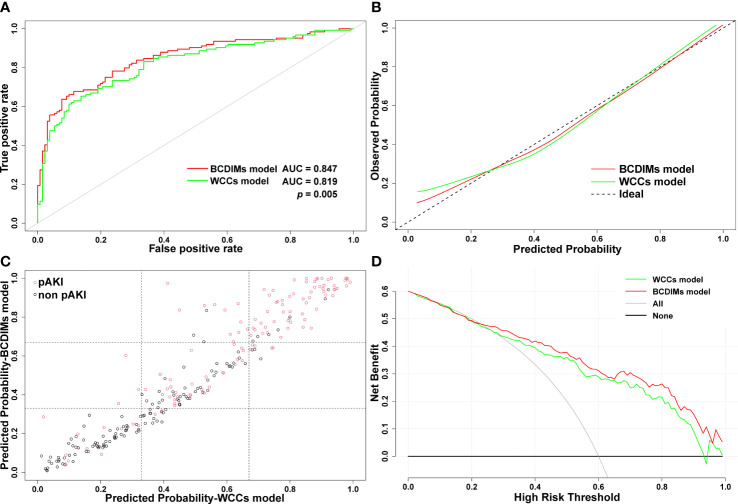
Comparison of the performance of BCDIMs model and WCCs model. **(A)** ROC curves and the AUC of BCDIMs model and WCCs model. **(B)** The calibration plot of BCDIMs model and WCCs model. **(C)** The reclassification plot of BCDIMs model and WCCs model. The black circles represent patients without pAKI, and red circles represent patients with pAKI. **(D)** The decision curve of BCDIMs model and WCCs model. BCDIMs, blood count-derived inflammatory markers; WCCs, white cell counts; ROC, receiver operating characteristic; AUC, area under ROC curve; pAKI, postoperative acute kidney injury.

The AUC value of the BCDIMs model was 0.847 (95% confidence interval (CI): 0.799–0.895), which indicated good discriminating ability, and was significantly higher than the AUC value of the WCCs model (0.819, 95% CI: 0.767–0.872; *p* = 0.005) (**Figure 4A**; **Table 4**). The calibration plots (**Figure 4B**) and Hosmer–Lemeshow test (BCDIMs model: Hosmer–Lemeshow chi-square = 12.823, *p* = 0.118; WCCs model: Hosmer–Lemeshow chi-square = 11.987, *p* = 0.152) suggested that both the BCDIMs model and WCCs model were well calibrated. Then, we further calculated the NRI and IDI to analyze the enhancement effect of the BCDIMs model on the predictive ability compared to the WCCs model. The reclassification plot and reclassification tables of the BCDIMs and WCCs models are displayed in **Figure 4C**; **Supplementary Table 4**, and **Supplementary Table 5**. The NRI of the BCDIMs model compared to the WCCs model was 0.156 (95% CI: −0.016 to 0.260; *p* = 0.001) (**Table 4**), which suggested a significant enhancement effect of the BCDIMs model on the predictive ability. The result of the IDI of the BCDIMs model compared to the WCCs model was 0.067 (95% CI: 0.045–0.090; *p* < 0.001) (**Table 4**), which indicated a significant improvement in the predictive ability of the BCDIMs model compared to the WCCs model. Finally, DCA was applied to evaluate the clinical utility of the two models. The results revealed that compared to the WCCs model, more clinical net benefits could be obtained across almost the whole range by the BCDIMs model (**Figure 4D**). In summary, the BCDIMs model had good discriminating ability, predictive ability, and clinical utility, and the performance of the BCDIMs model was significantly better than that of the WCCs model.

**Table 4:** Discriminating and predictive ability of different models.

Model	AUC (95% CI)	*p*-Value for AUC	NRI (95% CI)	*p*-Value for NRI	IDI (95% CI)	*p*-Value for IDI
WCCs model	0.847 (0.799–0.895)	**0.005**				
BCDIMs model	0.819 (0.767–0.872)		0.156 (−0.016 to 0.260)	**0.001**	0.067 (0.045–0.090)	**<0.001**

p-Value in bold indicates that p-value is less than 0.05.

WCCs, white cell counts; BCDIMs, blood count-derived inflammatory markers; AUC, area under curve; NRI, net reclassification index; IDI, integrated discrimination improvement; CI, confidence interval.

### Validating the relationship between the MLR and pAKI, and the predictive ability of the MLR for pAKI using data from the MIMIC-IV database

After patient data were extracted from the MIMIC-IV database, we found that only the MLR was significantly increased in patients from the pAKI group (**Supplementary Figure 2**). Considering the incomplete records of the operative variables in the MIMIC-IV database, we selected age, gender (male), BMI, and MLR to construct a predictive model for pAKI (BCDIMs-MIMIC model, **Table 5**). Similarly, age, gender (male), BMI, monocytes, and lymphocytes were selected to construct another predictive model for pAKI (WCCs-MIMIC model, **Supplementary Table 6**). According to the results of the logistic regression, MLR was an independent risk factor for pAKI (**Table 5**).

**Table 5:** Logistic regression model for postoperative AKI using data from MIMIC-IV database (BCDIMs-MIMIC model).

Variables	β	OR	95% CI	*p*-Value
Age	0.044	1.045	0.979–1.115	0.185
Gender (male)	−0.071	0.932	0.174–4.977	0.934
BMI	0.082	1.085	0.964–1.222	0.178
MLR	3.158	23.522	1.509–366.644	**0.024**

p-Value in bold indicates that p-value is less than 0.05.

AKI, acute kidney injury; BCDIMs, blood count-derived inflammatory markers; BMI, body mass index; MLR, monocyte–lymphocyte ratio.

**Figure 5:**
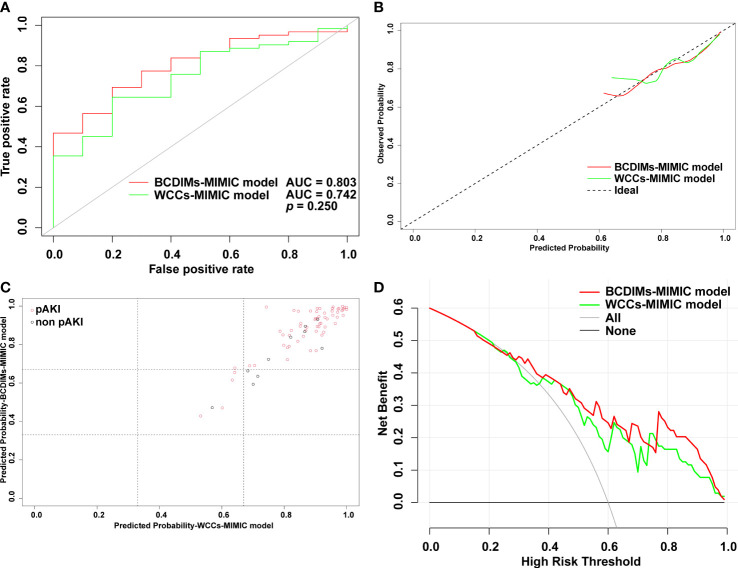
Comparison of performance of BCDIMs-MIMIC model and WCCs-MIMIC model. **(A)** ROC curve and the AUC of BCDIMs-MIMIC model and WCCs-MIMIC model. **(B)** The calibration plot of BCDIMs-MIMIC model and WCCs-MIMIC model. **(C)** The reclassification plot of BCDIMs-MIMIC model and WCCs-MIMIC model. The black circles represent patients without pAKI, and red circles represent patients with pAKI. **(D)** The decision curve of BCDIMs-MIMIC model and WCCs-MIMIC model. BCDIMs, blood count-derived inflammatory markers; WCCs, white cell counts; MIMIC, the Medical Information Mart for Intensive Care; ROC, receiver operating characteristic; AUC, area under ROC curve; pAKI, postoperative acute kidney injury.

The performance of the two models was also compared using the methods mentioned above. First, there was no significant difference in the AUC value between the BCDIMs-MIMIC model and WCCs-MIMIC model (0.803 *vs.* 0.742, *p* = 0.250) (**Figure 5A**). The Hosmer–Lemeshow test suggested that both models were well calibrated (BCDIMs-MIMIC model: Hosmer–Lemeshow chi-square = 2.698, *p* = 0.952; WCCs-MIMIC model: Hosmer–Lemeshow chi-square = 6.294, *p* = 0.614). The calibration plot also revealed that both models were well-calibrated (**Figure 5B**). The reclassification plot and reclassification tables of the BCDIMs-MIMIC model and the WCCs-MIMIC model are displayed in **Figure 5C**; **Supplementary Table 7**, and **Supplementary Table 8**. In addition, the results of the NRI demonstrated that the BCDIMs-MIMIC model could significantly enhance predictive ability when compared to the WCCs-MIMIC model (NRI = 0.316, 95% CI: −0.612 to 0.457, *p* = 0.030) (**Table 6**). Similarly, the IDI of the BCDIMs-MIMIC model compared to the WCCs-MIMIC model was 0.047 (95% CI: 0.006–0.088; *p* = 0.025) (**Table 6**), which indicated a significant improvement in the predictive ability of the BCDIMs-MIMIC model compared to the WCCs-MIMIC model. Finally, the results of the DCA revealed that compared to the WCCs-MIMIC model, more clinical net benefits could be obtained across almost the whole range by the BCDIMs-MIMIC model (**Figure 5D**). In summary, the BCDIMs-MIMIC model had good discriminating ability, predictive ability, and clinical utility, and the performance of the BCDIMs-MIMIC model was significantly better than that of the WCCs-MIMIC model.

**Table 6:** Discriminating and predictive ability of different models.

Model	AUC (95% CI)	*p*-Value for AUC	NRI (95% CI)	*p*-Value for NRI	IDI (95% CI)	*p*-Value for IDI
WCCs-MIMIC model	0.803 (0.679–0.927)	0.250				
BCDIMs-MIMIC model	0.742 (0.597–0.887)		0.316 (−0.612 to 0.457)	**0.030**	0.047 (0.006–0.088)	**0.025**

p-Value in bold indicates that p-value is less than 0.05.

MIMIC-IV database, the Medical Information Mart for Intensive Care IV database; AUC, area under the curve; WCCs, white cell counts; BCDIMs, blood count-derived inflammatory markers; NRI, net reclassification index; IDI, integrated discrimination improvement; CI, confidence interval.

### The relationship between the MLR and survival of patients who underwent TAAD surgery

**Figure 6:**
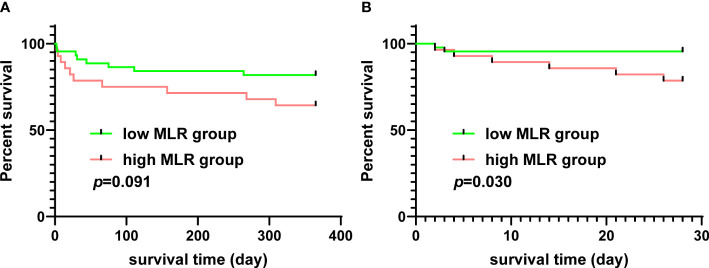
The relationship between MLR and survival of patients who underwent TAAD surgery from the MIMIC-IV database. **(A)** The 1-year Kaplan–Meier curves of patients with a high or low MLR. **(B)** The 28-day Kaplan–Meier curves of patients with a high or low MLR. MLR, monocyte–lymphocyte ratio; TAAD, type A aortic dissection; MIMIC-IV database, the Medical Information Mart for Intensive Care (MIMIC)-IV database.

The relationship between the MLR and the survival of patients who underwent TAAD surgery was analyzed using data from the MIMIC-IV database. The mean MLR of patients extracted from the MIMIC-IV database was 0.678, and patients were divided into the low MLR group and high MLR group using the mean MLR as the threshold. The 1-year survival rate of patients with a high MLR was lower than in patients with a low MLR; however, this did not reach statistical significance (**Figure 6A**). The 28-day survival rate of patients with a high MLR was significantly lower than in patients with a low MLR (**Figure 6B**).

## Discussion

In this study, the data of patients who underwent TAAD surgery from our center and the MIMIC-IV database were analyzed. The results showed that the MLR was an independent risk factor for pAKI. The predictive model based on BCDIMs had good discriminating ability, predictive ability, and clinical utility. Moreover, the performance of the BCDIMs model was better than the model based on corresponding WCCs. The MLR was also found to be significantly associated with the 28-day survival of patients who underwent TAAD surgery. The present study revealed that the MLR could be a significant predictor of pAKI and survival in patients who underwent TAAD surgery.

TAAD involves the ascending aorta, and patients with TAAD require swift surgery to save their lives (1). The in-hospital mortality of patients who underwent TAAD surgery is 27% according to the IRAD study in 2009 (6). According to Silaschi et al., the 30-day mortality of patients who underwent TAAD surgery was 7.1% in the hands of an experienced surgeon (5). In this study, the in-hospital mortality of patients who underwent TAAD surgery was 3.53%, which was lower than previous reports. The most important reason for the difference in mortality was that some of the patients who died in the hospital were excluded from this study because of missing data.

Postoperative complications, such as AKI, ischemia, and pneumonia, can significantly affect the prognosis of patients (21). AKI is a common but serious complication after TAAD surgery; the incidence of AKI after TAAD ranges from 20.2% to 66.7% (22–24). The large difference in the incidence reported in previous studies might be due to the different study populations and the different definitions of AKI. In this study, AKI was defined according to the criteria of KDIGO, which was designed to diagnose AKI in the early stage (17). The incidence of pAKI in this study was 48.63%, which was close to that reported by Chen et al. (25) and Guan et al. (26). Arnaoutakis et al. analyzed data from the IRAD database and reported that patients with pAKI had higher in-hospital mortality and a higher incidence of other postoperative complications, such as cerebrovascular accident and prolonged ventilation (27). Moreover, the 5-year survival rate of patients with pAKI was significantly decreased (27). Wang et al. reported that pAKI was significantly associated with poor long-term survival in elderly patients who underwent TAAD surgery (11). Our study had similar findings that patients with pAKI had higher rates of in-hospital mortality, cerebral complications, and hepatic dysfunction. Furthermore, data from the MIMIC-IV suggested that pAKI was significantly associated with a prolonged ventilation time and a poor 1-year survival rate. Considering the relationship between pAKI and the poor prognosis of patients who underwent TAAD surgery, further exploration of readily available predictors and predictive models is needed to predict pAKI at an early stage.

Inflammation contributes to the development and progression of AKI. The inflammatory response can lead to dysregulation of renal blood vessels and impair renal function (28, 29). Inflammatory cytokines, such as tumor necrosis factor-α (TNF-α) and interleukin-6 (IL-6), induce infiltration of immune cells in the renal parenchyma, cause cell injury, and result in impairment of renal function (28, 29). Thus, we speculated that preoperative inflammatory markers might be good predictors for pAKI. BCDIMs, including the NLR, MLR, PLR, SII, and SIRI, can reflect the systemic inflammation status of patients (12). Studies have demonstrated that BCDIMs were significantly associated with cardiovascular diseases. Ma et al. found that the MLR was significantly correlated with the incidence, number, and echo characteristics of carotid plaques in patients with coronary artery disease (30). Tamaki et al. reported that the combination of the NLR and PLR could be a novel predictor for cardiac death in patients with decompensated heart failure (31). Zhu et al. revealed that elevation of the preoperative NLR was related to the short-term adverse outcomes of patients with type B AD (32). On the other hand, the BCDIMs were more readily available, time-saving, and inexpensive than other inflammatory markers, such as C reactive protein, TNF-α, and IL-6.

In the present study, the data of patients from our center were analyzed, and the predictive model based on BCDIMs was constructed. The logistic regression analysis indicated that the MLR, BMI, and CA minimum temperature were independent risk factors for pAKI. Other variables in the predictive model included smoking status (smoker), NLR, total arch replacement, operation time, and aortic cross-clamp time. Similarly, the study of Li et al. suggested that BMI was an independent risk factor for pAKI of patients who underwent TAAD (33). Hypothermia is an important technique in TAAD surgery, which could protect the nervous system and other organs from ischemic injury by decreasing cellular consumption and slowing cellular metabolism (34). However, there were few studies that focused on the relationship between CA minimum temperature and pAKI and need further investigation. The operation time in patients who underwent total arch replacement was significantly longer than that of patients who underwent semi-arch replacement (452.41 ± 114.59 *vs.* 343.42 ± 81.56 min, *p* < 0.001, **Supplementary Figure 3**). A longer operation time is usually accompanied by a longer cardiopulmonary bypass time, and both the operation and cardiopulmonary bypass can trigger an inflammatory response and oxidative stress, both of which could lead to AKI (28). After the construction of the predictive model, methods including the ROC curve, calibration plot, NRI, IDI, and DCA were utilized to evaluate the performance of the model. The results suggested that the BCDIMs model had good discriminating ability, predictive ability, and clinical utility. We also constructed the WCCs model by replacing the MLR and NLR with corresponding counts of monocytes, neutrophils, and lymphocytes. After comparing the performance of the two models, we found that the performance of the BCDIMs model was moderately better than that of the WCCs model, which reached statistical significance. Thereafter, data from the MIMIC-IV database further validated these results. Moreover, patients with a high MLR had a significantly poorer 28-day survival rate when compared to patients with a low MLR. The MLR reflects the levels of systemic inflammation and immune response activation of patients (35). Elevation of the MLR represents the imbalance in innate and adaptive immunity (35). Briefly, when exposed to a stressor, monocytes accumulate and differentiate into inflammatory dendritic cells and macrophages and then activate the proinflammatory response (35, 36). In addition, proinflammatory activation of monocytes could result in oxidative stress in endothelial cells (36). The inflammatory response and oxidative stress initiated by monocytes might impair renal function and eventually lead to AKI. Lymphocytes can regulate the phenotype of monocytes (30), but the exact role of lymphocytes in pAKI remains unclear and needs further exploration.

With the predictive model, pAKI could be predicted and prevented at an early stage. As mentioned above, inflammatory response contributes to the development pAKI. Jang et al. reported that suppression of inflammatory response using specific antibodies could significantly ameliorate kidney injury in the AKI animal model (37). In clinical practice, Milne et al. suggested that angiotensin-converting enzyme inhibitors (ACEIs) should be stopped early before cardiac surgery to prevent pAKI (38), as ACEIs might be associated with intraoperative hypotension and vasodilatory shock after cardiopulmonary bypass (39, 40). A prospective randomized controlled study conducted by Kamenshchikov et al. demonstrated that nitric oxide delivery during cardiopulmonary bypass reduced pAKI (41). In terms of non-cardiac surgery, Mita et al. reported that strict blood glucose control by an artificial endocrine pancreas during hepatectomy could prevent pAKI (42); Shelby et al. suggested that optimizing perioperative fluid management in patients who underwent complex abdominal wall reconstruction could prevent pAKI (43). However, more effective strategies to protect patients who underwent TAAD surgery from pAKI need further exploration.

Similarly, Ma et al. (44) (study population: n = 190) and Chen et al. (45) (study population: n = 159) reported that the lymphocyte–monocyte ratio (LMR) was negatively associated with pAKI in patients who underwent TAAD surgery. Consistent with our study, their studies showed that the MLR had predictive value for pAKI. However, there were obvious differences between our study and the studies of Ma et al. (44) and Chen et al. (45) First, the study population of our study was larger than studies of Ma et al. (44) and Chen et al. (45). Moreover, data from the MIMIC-IV database were further used to validate the role of the MLR in patients who underwent TAAD surgery. A larger study population and validation of an external dataset could improve the accuracy of the analysis. Second, methods including the ROC curve, calibration plot, NRI, IDI, and DCA were utilized to evaluate the performance of the model, which estimated more dimensions of the model performance. Third, we compared the performance of the models based on BCDIMs and WCCs using the methods mentioned above to further elucidate the predictive value of the MLR for pAKI. Fourth, the relationship between the MLR and the survival of patients who underwent TAAD surgery was assessed. In summary, our study evaluated the role of the MLR in pAKI more comprehensively.

The main limitation was that our study only included patients from a single center. In the future, a multi-center study is needed to further elucidate the role of the MLR in pAKI of patients who underwent TAAD. However, we did use data from the MIMIC-IV database to validate the findings in this study, which could reduce this weakness to some extent. In addition, because of the lack of urine output data in our center, we did not use the urine output criteria of KDIGO to define AKI; only the SCr criteria were used. Similarly, there were previous studies that only used the SCr criteria of KDIGO to define AKI (11, 33).

## Conclusion

Our study suggested that the MLR was an independent risk factor for pAKI. A predictive model based on BCDIMs was constructed and had good discriminating ability, predictive ability, and clinical utility. Moreover, the performance of the BCDIMs model was moderately better than that of the WCCs model, which reached statistical significance. Finally, a high MLR was significantly associated with poor short-term survival of patients who underwent TAAD surgery.

## Data availability statement

The original contributions presented in the study are included in the article/**Supplementary Material**. Further inquiries can be directed to the corresponding author.

## Ethics statement

The study was conducted in accordance with the “Declaration of Helsinki” and the Ethics Committee of Xiangya Hospital, Central South University approved this study. Written informed consent was waived because of the observational, retrospective nature of this study.

## Author contributions

YC and KD extracted data from the MIMIC-IV database, analyzed the data, completed figures, and wrote the manuscript. CF, HS, and WL collected data from our center, processed the raw data, and completed the tables. C-eT and FL designed the research. FL reviewed and edited the manuscript. All authors contributed to the article and approved the submitted version.
