# Supplementary material for: The predictive values of monocyte–lymphocyte ratio in postoperative acute kidney injury and prognosis of patients with Stanford type A aortic dissection
Source: Front Immunol. 2023 Jul 24;14:1195421. doi: 10.3389/fimmu.2023.1195421 (PMC10404983; doi:10.3389/fimmu.2023.1195421)
Supplement: Supplementary file 1 [file DataSheet_1.docx]

| Variables | β | OR | 95% CI | *p* value |
| --- | --- | --- | --- | --- |
| MLR | 2.528 | 12.533 | 6.163-27.880 | <0.001 |
| SIRI | 0.188 | 1.207 | 1.143-1.284 | <0.001 |
| NLR | 0.119 | 1.127 | 1.079-1.181 | <0.001 |
| Preoperative monocyte | 1.441 | 4.226 | 2.184-8.614 | <0.001 |
| Operation time | 0.005 | 1.005 | 1.002-1.007 | <0.001 |
| Preoperative lymphocyte | -0.998 | 0.369 | 0.215-0.607 | <0.001 |
| SII | 0.0004 | 1.0004 | 1.0002-1.0006 | <0.001 |
| BMI | 0.113 | 1.119 | 1.050-1.198 | <0.001 |
| Preoperative neutrophil | 0.097 | 1.102 | 1.032-1.181 | 0.004 |
| PLR | 0.003 | 1.003 | 1.001-1.005 | 0.005 |
| Gender (male) | 0.756 | 2.129 | 1.249-3.680 | 0.006 |
| Preoperative WBC | 0.081 | 1.084 | 1.020-1.155 | 0.011 |
| CA minimum temperature | -0.124 | 0.884 | 0.800-0.971 | 0.011 |
| Cardiopulmonary bypass time | 0.005 | 1.005 | 1.001-1.009 | 0.014 |
| Total arch repair | 0.838 | 2.311 | 1.131-4.968 | 0.025 |
| Aortic cross clamp time | 0.005 | 1.005 | 1.001-1.010 | 0.027 |
| Platelet usage intraoperation | 0.327 | 1.386 | 1.016-1.915 | 0.042 |
| Smoking | 0.502 | 1.652 | 1.007-2.725 | 0.048 |

Supplementary Table 1: Univariate analysis of risk factors for postoperative AKI

Abbreviations: AKI: acute kidney injury, MLR: monocyte-lymphocyte ratio, SIRI: systemic inflammatory reaction index, NLR: neutrophil-lymphocyte ratio, SII: systemic inflammatory index, BMI: body mass index, PLR: platelet-lymphocyte ratio, WBC: white blood cell, CA: circulatory arrest.

Supplementary Table 2: Variance inflation factors of variables in predictive model

| Variables | Variance inflation factor |
| --- | --- |
| Gender | 1.513114 |
| Age | 1.249985 |
| BMI | 1.243512 |
| Smoking | 1.208646 |
| SIRI | 9.410002 |
| SII | 16.602117 |
| NLR | 2.907336 |
| MLR | 7.531755 |
| PLR | 12.397748 |
| CA minimum temperature | 1.379216 |
| Total arch repair | 1.501927 |
| platelet usage intraoperation | 1.274424 |
| Operation time | 3.828434 |
| Cardiopulmonary bypass time | 3.973611 |
| Aortic cross clamp time | 1.654300 |

Abbreviations: BMI: body mass index, SIRI: systemic inflammatory response index, NLR: neutrophil-lymphocyte ratio, MLR: monocyte-lymphocyte ratio, PLR: platelet-lymphocyte ratio.

| Variables | β | OR | 95% CI | *p* value |
| --- | --- | --- | --- | --- |
| BMI | 0.106 | 1.111 | 1.028-1.202 | **0.008** |
| Smoking (smoker) | 0.550 | 1.734 | 0.954-3.151 | 0.071 |
| Neutrophil | 0.016 | 1.016 | 0.928-1.113 | 0.730 |
| Lymphocyte | -1.665 | 0.189 | 0.097-0.369 | **<0.001** |
| Monocyte | 2.180 | 8.844 | 3.238-24.154 | **<0.001** |
| Total arch repair (yes) | 0.629 | 1.876 | 0.700-5.028 | 0.211 |
| CA minimum temperature | -0.199 | 0.820 | 0.719-0.934 | **0.003** |
| Operation time | 0.002 | 1.002 | 0.998-1.005 | 0.371 |
| Aortic cross clamp time | 0.003 | 1.003 | 0.998-1.008 | 0.291 |

Supplementary Table 3: Logistic regression model for postoperative AKI (WCCs model)

Abbreviations: AKI: acute kidney injury, BMI: body mass index, CA: circulatory arrest. *p* value in bold indicates that *p* value is less than 0.05.

Supplementary Table 4: Reclassification table for patients without pAKI using different models

| Predicted probability using WCCs model | Predicted probability using BCDIMs model | | | NRI value for patients  without pAKI |
| --- | --- | --- | --- | --- |
|  | <=0.33 | 0.33-0.67 | >=0.67 |  |
| <=0.33 | 66 | 0 | 0 | [13+3-(0+3)]/131  =0.0992 |
| 0.33-0.67 | 13 | 39 | 3 |  |
| >=0.67 | 0 | 3 | 7 |  |

Abbreviations: pAKI: postoperative acute kidney injury, WCCs: white cell counts, BCDIMs: blood count-derived inflammatory markers, NRI: net reclassification index.

Supplementary Table 5: Reclassification table for patients with pAKI using different models

| Predicted probability using WCCs model | Predicted probability using BCDIMs model | | | NRI value for patients  with pAKI |
| --- | --- | --- | --- | --- |
|  | <=0.33 | 0.33-0.67 | >=0.67 |  |
| <=0.33 | 13 | 3 | 0 | [3+11-(2+5)]/124  =0.0565 |
| 0.33-0.67 | 2 | 29 | 11 |  |
| >=0.67 | 0 | 5 | 61 |  |

Abbreviations: pAKI: postoperative acute kidney injury, WCCs: white cell counts, BCDIMs: blood count-derived inflammatory markers, NRI: net reclassification index.

| Variables | β | OR | 95% CI | *p* value |
| --- | --- | --- | --- | --- |
| Age | 0.038 | 1.039 | 0.976-1.106 | 0.232 |
| Gender (male) | 0.313 | 1.367 | 0.251-7.454 | 0.718 |
| BMI | 0.052 | 1.053 | 0.939-1.181 | 0.375 |
| Monocyte | 2.491 | 12.070 | 0.926-157.298 | 0.057 |
| Lymphocyte | -0.297 | 0.743 | 0.310-1.780 | 0.506 |

Supplementary Table 6: Logistic regression model for postoperative AKI using data from MIMIC-IV database (WCCs-MIMIC model)

Abbreviations: AKI: acute kidney injury, BMI: body mass index, WCCs: white cell counts. *p* value in bold indicates that *p* value is less than 0.05.

Supplementary Table 7: Reclassification table for patients without pAKI using different models based on data from MIMIC-IV database

| Predicted probability using WCCs-MIMIC model | Predicted probability using BCDIMs-MIMIC model | | | NRI value for patients  without pAKI |
| --- | --- | --- | --- | --- |
|  | <=0.33 | 0.33-0.67 | >=0.67 |  |
| <=0.33 | 0 | 0 | 0 | 3/10  =0.300 |
| 0.33-0.67 | 0 | 1 | 0 |  |
| >=0.67 | 0 | 3 | 6 |  |

Abbreviations: pAKI: postoperative acute kidney injury, MIMIC-IV database: the Medical Information Mart for Intensive Care IV database, WCCs: white cell counts, BCDIMs: blood count-derived inflammatory markers, NRI: net reclassification index.

Supplementary Table 8: Reclassification table for patients with pAKI using different models based on data from MIMIC-IV database

| Predicted probability using WCCs-MIMIC model | Predicted probability using BCDIMs-MIMIC model | | | NRI value for patients  with pAKI |
| --- | --- | --- | --- | --- |
|  | <=0.33 | 0.33-0.67 | >=0.67 |  |
| <=0.33 | 0 | 0 | 0 | 1/62  =0.016 |
| 0.33-0.67 | 0 | 4 | 1 |  |
| >=0.67 | 0 | 0 | 57 |  |

Abbreviations: pAKI: postoperative acute kidney injury, MIMIC-IV database: the Medical Information Mart for Intensive Care IV database, WCCs: white cell counts, BCDIMs: blood count-derived inflammatory markers, NRI: net reclassification index.


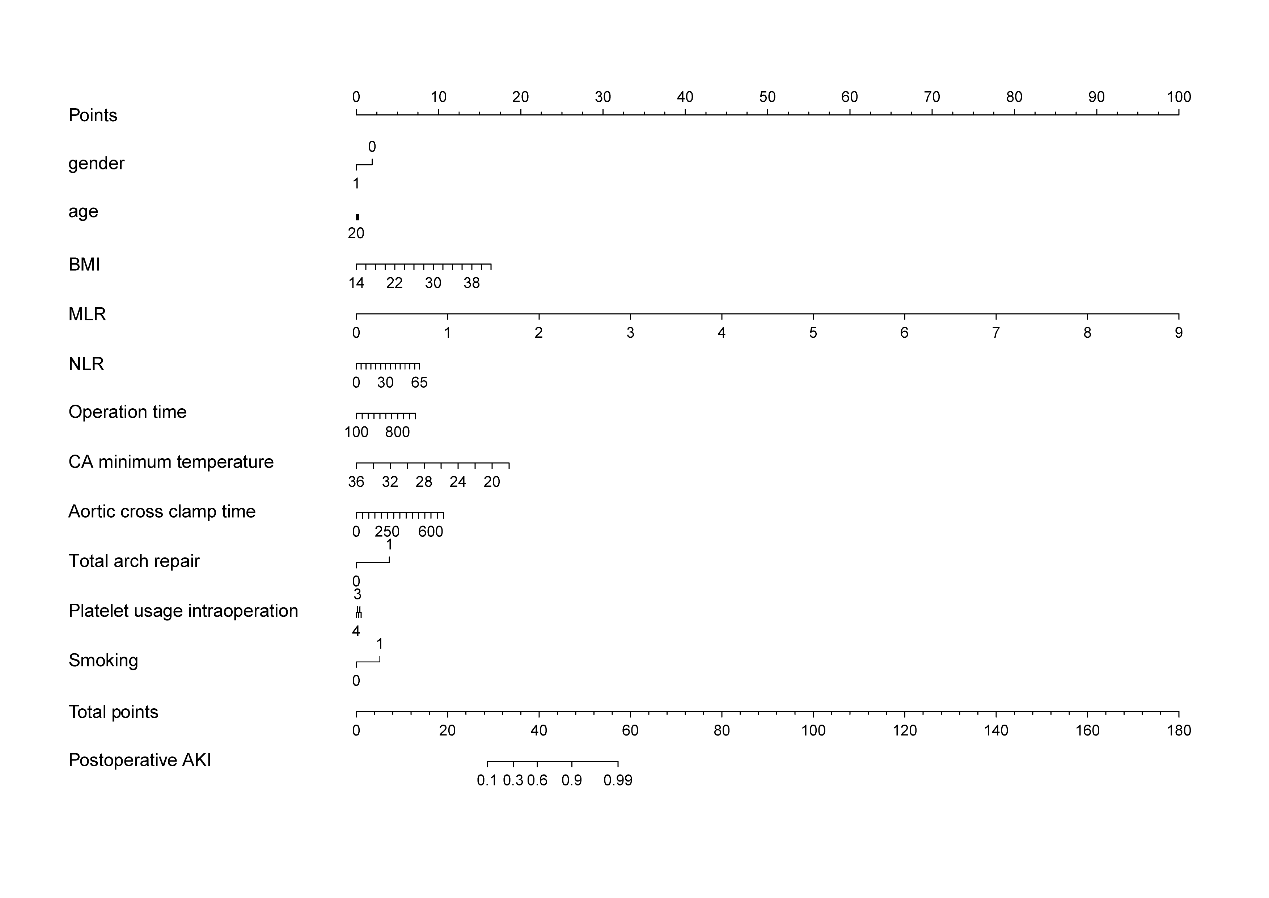


Supplementary Figure 1: The nomogram of predictive model including gender, age, and platelet usage intraoperation. In the nomogram, gender 0 represented female, gender 1 represented male, smoking 0 represented non-smoker, and smoking 1 represented smoker. Abbreviations: BMI: body mass index, NLR: neutrophil-lymphocyte ratio, MLR: monocyte-lymphocyte ratio, PLR: platelet-lymphocyte ratio, AKI: acute kidney injury.


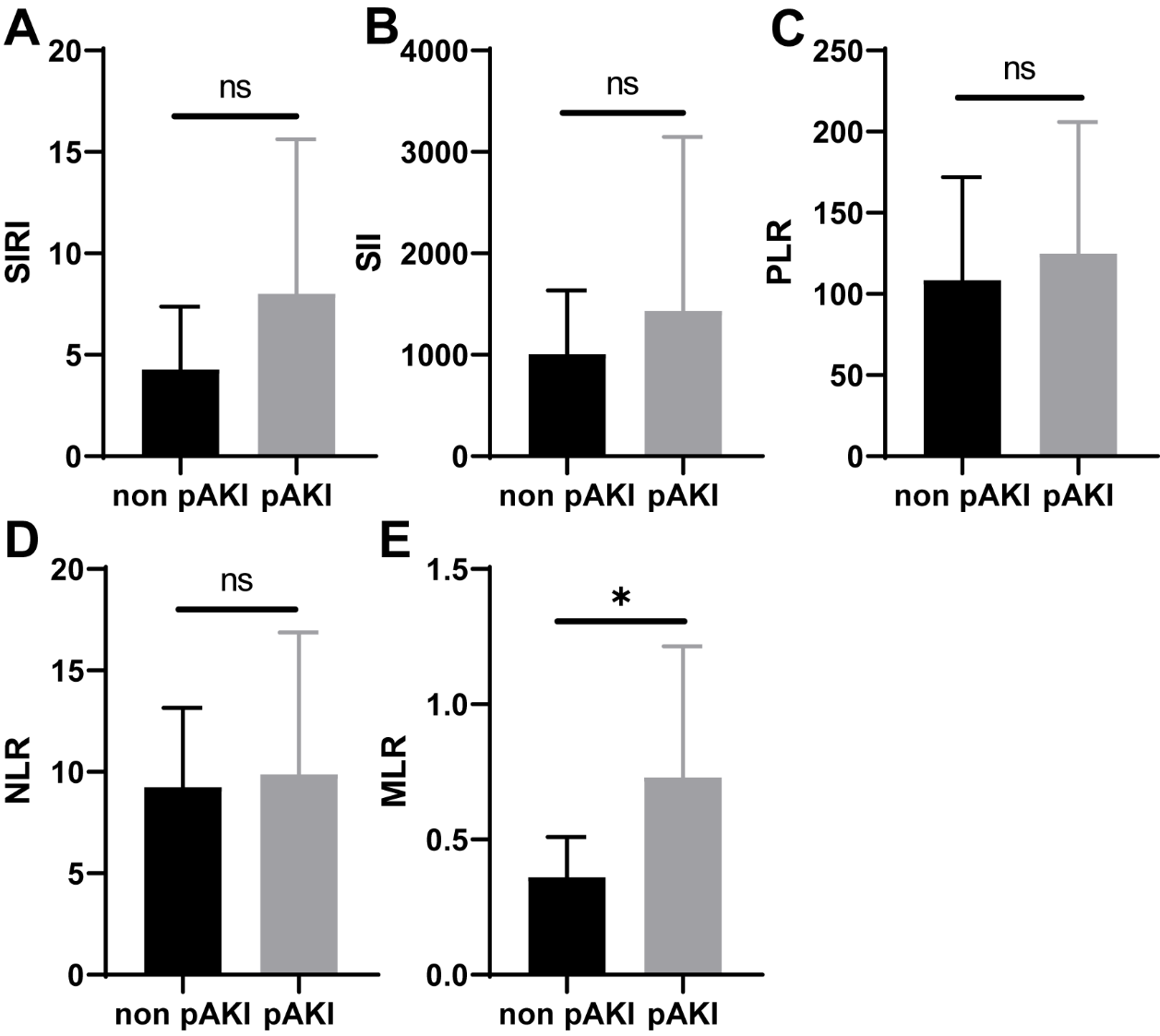


Supplementary Figure 2: The values of BCDIMs in patients with or without pAKI from MIMIC-IV database. A-E: The values of SIRI, SII, PLR, NLR, and MLR in patients with or without pAKI. Abbreviations: BCDIMs: blood count-derived inflammatory markers, MIMIC-IV database: the Medical Information Mart for Intensive Care (MIMIC)-IV database, pAKI: postoperative acute kidney injury, SIRI: systemic inflammatory response index, SII: systemic inflammatory index, PLR: platelet-lymphocyte ratio, MLR: monocyte-lymphocyte ratio, NLR: neutrophil-lymphocyte ratio. ns: non-significance, *, p value < 0.05.


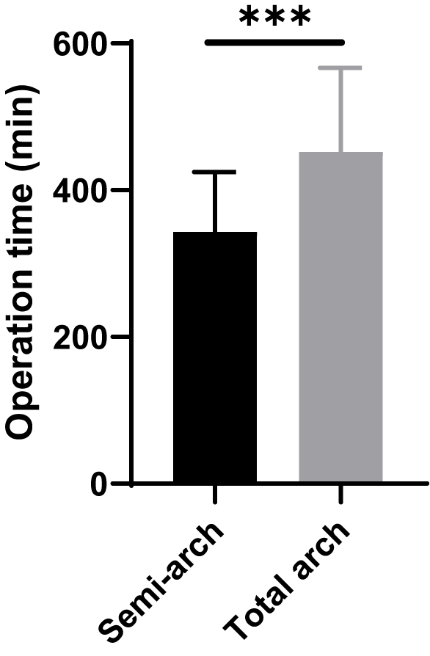


Supplementary Figure 3: The operation time of patients underwent semi-arch or total arch replacement. ***, p value < 0.001.
